# Aspects of Modern Biobank Activity – Comprehensive Review

**DOI:** 10.1007/s12253-018-0418-4

**Published:** 2018-05-05

**Authors:** Wiktor Paskal, Adriana M. Paskal, Tomasz Dębski, Maciej Gryziak, Janusz Jaworowski

**Affiliations:** 1 The Department of Histology and Embryology, Laboratory of Centre for Preclinical Research, Medical University of Warsaw, ul. Banacha 1B, 02-097 Warsaw, Poland; 2 Plastic Surgery Department, Centre of Postgraduate Medical Education, Warsaw, Poland; 3 The Department of Applied Pharmacy, Medical University of Warsaw, Warsaw, Poland; 4 Maria Sklodowska-Curie Institute of Oncology, Warsaw, Poland

**Keywords:** Biobank, Personalized medicine, Biorepository, Biospecimen, Tissue Banking

## Abstract

Biobanks play an increasing role in contemporary research projects. These units meet all requirements to regard them as a one of the most innovative and up-to-date in the field of biomedical research. They enable conducting wide-scale research by the professional collection of biological specimens and correlated clinical data. Pathology units may be perceived roots of biobanking. The review aims at describing the concept of biobanks, their model of function and scientific potential. It comprises the division of biobanks, sample preservation methods and IT solutions as well as guidelines and recommendations for management of a vast number of biological samples and clinical data. Therefore, appropriate standard operating procedures and protocols are outlined. Constant individualization of diagnostic process and treatment procedures creates the niche for translational units. Thus, the role of biobanks in personalized medicine was also specified. The exceptionality of biobanks poses some new ethical-legal issues which have various solutions, in each legal system, amongst the world. Finally, distribution and activity of European biobanks are mentioned.

## Introduction - Concept of Biobanks

Immense development in the field of biomedical research multiplies challenge in the eternal problem of obtaining, preserving and analyzing human samples. Ever since the first experiment had been conducted, a problem of preserving samples for future purposes appeared. Furthermore, the possibility of harvesting biological material during routine activities of medical and biological professionals was the trigger to manage it in an organized manner. Hospital pathology units are limited in storing a large number of preserved samples. Also, they are primarily designed to diagnose the obtained specimens, what implies restricted samples’ legal status (analyses limited to diagnostic purposes, not for research) and certain collection method. Thus, came up a niche for a novel solution – biobanks (BBs).

## Definition and Genesis of Biobanks

Wide heterogeneity and still developing central control over such initiatives causes difficulty to give one and sufficient definition. Principally, biobanks are professional repositories of biologic samples. Activities in the scope of a biobank include: storing specimens harvested over time, combined with clinical, epidemiological and general source data. Moreover, collected samples are processed, primarily analyzed and adequately preserved for sharing with the wider scientific community. They also deliver details about the acquisition, handling and storage of each sample – e.g. ischemia time, preservation method, shipping conditions. Summary of definitions of human biobanks and relevant terms are well described by Fransson et al. [1]

Biobank workflow is maintained in a strictly organized manner. Standard operating procedures (SOPs) ensure correct implementation of essential biobanking components (samples’ donors’ anonymization, samples: acquisition, transport, preparation and analysis process faultlessness, proper storage conditions and terms of samples’ sharing (e.g. local/international law)). For these reasons, biobanks combine a wide variety of data mainly for research purposes.

One of the very first attempt to create a detached unit responsible for human specimen storage has begun with Framingham Heart Study (FHS) established to collect blood samples and patient data since 1948 [2]. Construction of the study created a unique opportunity to combine biological data obtained via blood analysis with clinical examinations and lifestyle interviews to elicit risk factors for cardiovascular disorders. However, the beginning of biospecimen repositories may be counted since hospital pathology units started to store harvested samples. So that, the history of BBs is longer than it appears [3].

### Types of Biobanks

**Fig. 1:**
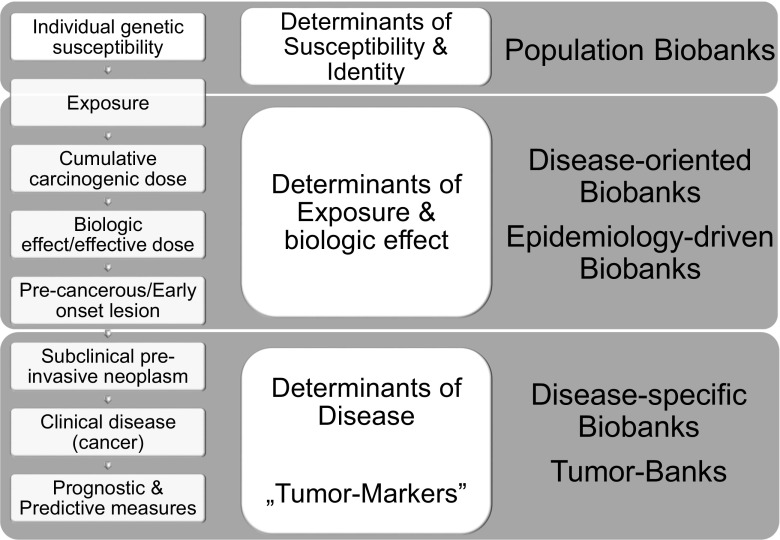
Types of biobanks based on type of biomarker research. The different stage of the natural history of a disease (left column) defines types of biomarkers that can be utilized (second column). Finally, biobank’s type originates from the biomarker classification (right column). [4, 5]

The need for correlating a wide variety of data gave rise to particular types of biorepositories, namely: population-based, disease-oriented, and tissue biobanks (See Fig. 1) [1, 4–6].

Population-based units comprise samples and epidemiological/clinical data collected from volunteers without specific inclusion or exclusion criteria – material aims to mirror the status of the general population [7]. One of the best example of this type is population DNA biobank, which was established in order to examine human genome within PHG (Public Health Genomics) projects like HuGENet™, Estonian Biobank in Estonian Genome Center, CARTaGENE [8–10]. For instance, FarGen Project aims at sequencing whole genomes of Faroese residents to obtain more thorough public health data (http://www.fargen.fo, date of access: 27.07.2017). Mentioned BBs not only collect DNA or molecular data, but also combine various clinical data (physical examination, demographic, laboratory test results, miscellaneous questionnaire data etc.). The results of the first human genome sequencing enabled the development of next generation sequencing techniques (NGS). It has enormous influence on biobanks since it decreases the cost and time of analysis per sample and produces complete information about each patient’s genome, epigenome, transcriptome and even more. Samples of specific tissue types are the source of data about the public health status. Cervical cytology biobanks are an example of such units [11].

Many biobanks are established in relation with particular scientific projects. “Children of the 90s” study led to collecting a great amount of placenta tissue from 14,541 pregnant women between 1990 and 1992 in South West England [12]. Population-based biobanks are the source of samples for many research groups. The strength and importance of such studies are enormous thanks to the large-scale character [13–16].

Setting inclusion or exclusion criteria during collection of samples leads to disease-oriented biobanks (or disease-oriented biobanks for epidemiology). The aim is to determine specific exposure factors for a disease by the careful collection of specimen from a patient, often comprising various samples from the subject. For instance, HiWATE (Health impacts of long-term exposure to disinfection byproducts in drinking water) project focuses on determining risks for human health that are brought about byproducts in drinking water. Studies require a wide range of specimens (e.g. semen, biomarkers in the blood) [4–6, 11, 17, 18]. In the EPIC (European Prospective Investigation into Cancer and Nutrition) associated studies BBs play the role as a source of data for e.g. IGF-I role in ovarian cancer development, fish consumption and mortality and lifestyle influence on breast cancer risk [5, 19–21]. BBs also solve a problem of gathering a significant number of samples from the patients with rare diseases. MRC Centre Biobank for Neuromuscular Diseases in Newcastle and London contributed to diagnostics, basic science research, industry, drug development, and therapy of neuromuscular diseases [22]. Disease-oriented biobanks gather also materials collected from patients suffering from infectious diseases. The UCSF ASB (AIDS Specimen Biobank) collects peripheral blood mononuclear cells (PBMC) that contributed to the discovery of factors that cause AIDS and Kaposi’s sarcoma [23]. Recent Ebola outbreak in Africa led WHO to establish new initiative-Ebola Biobank. 100,000 samples of blood, semen, urine and breast milk from confirmed and suspected Ebola patients will be stored and analyzed to increase understanding and predicting Ebola outbreaks [24].

Finally, the tissue biobanks, known also as a tumor banks, aim at exploring the biology of a particular sample by collecting and comparing unaffected tissues and the neoplastic one. Generally, the main purpose is to deepen the knowledge about the molecular basis of the disease and/or determine new applicable biomarkers of an examined disorder. These projects require isolation of intracellular particles such as DNA, RNA and proteins. Ultimately, obtained biological data from high-throughput analyses are combined and correlated with clinical data [4, 5]. To exemplify, following projects are worth mentioning: European Human Tumor Frozen Tissue Bank, the National Cancer Institute Office of Biorepositories and Biospecimen Research, the Canadian Tumor Repository Network (CTRNet) [4, 5].

## Basic Components of Biobank

European Commission Joint Research Centre (EC-JRC) provides the community with general feature of biobanks such as [6, 11]:

- collection and storage of biological materials combined with medical, epidemiological data,
- dynamic development of the biobanks - continuous collection in a long-term prospect,
- association with an ongoing research project,
- application of specimens’ anonymization for the donors’ privacy sake,
- implementation of governance standards and procedures.

Given the exact purpose of a biobank establishment, its characteristics vary for the best suitability to a project (e.g. tissue type, a target of the study).

**Fig. 2:**
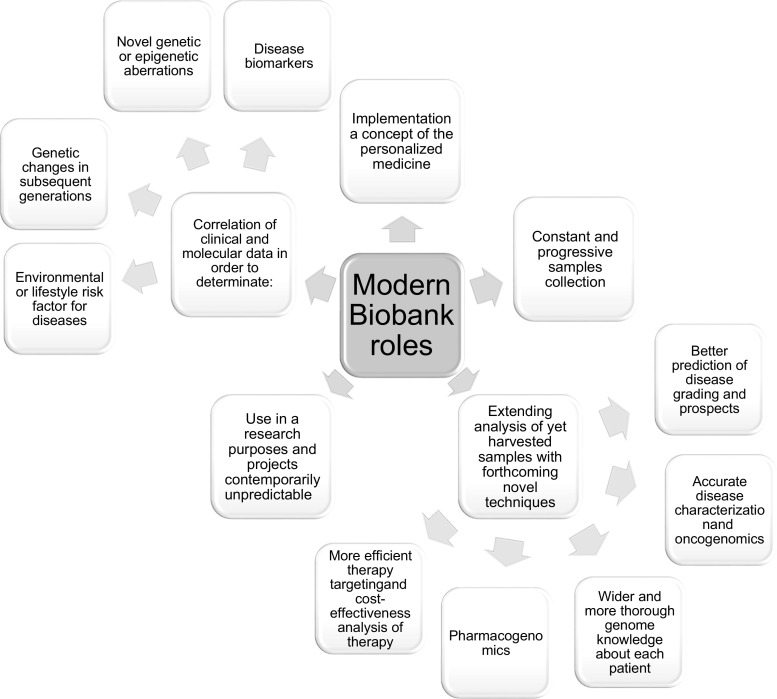
Biobanks role scheme [3, 25]

A combination of professional biological storage solutions, novel bioinformatics data processing systems and firm governance compose a model of biobank for multiple purposes (see Fig. 2). Samples from the model BB are prepared for basic research, drug development and other yet unplanned research on the date of samples collection.

### Samples Analysis and Preservation Methods

Sample acquisition process starts with obtaining informed consent from the patient (see more 3.3 Ethical and legal issues). Sample’s cycle in biobank starts with harvesting the sample during medical procedures. In the case of tumor biobanks, the research purposes are not allowed to interfere with diagnostic pathway (see Tab 2. MMI). Thus, involving a pathologist into the acquisition of specimen for biobanking may perfectly combine both diagnostic purposes with a proper biobanking harvest (avoiding tumor margin, necrosis, blood clots, irrelevant surrounding tissue). Care must be taken to place the sample, in a sterile manner, in the vial with appropriate media/buffers depending on planned analysis (see Tab. 1). Detached and/or thoroughly-trained staff is recommended. Samples should be shipped under strictly determined and monitored conditions (e.g. temperature, warm/cold ischemia time, maximum transport time). After the arrival at the Biobank unit, a sample is anonymized, labeled and handled to storage/analysis unit. Here, a crucial step – aliquoting is implemented. It comprises the division of sample for a smaller portion which decreases freeze/thaw cycles of a sample of a patient, creates a backup, eases the sharing of samples without thawing. In the UK Biobank, the standard aliquoting procedure for blood and urine from each patient produces 19 aliquots from 5 primary samples [55]. After completing survey data acquisition, the record is ready to be used in a project or shared. This multi-step process requires standardization to keep reproducibility. For this reason, Standard Operating Procedures (SOPs) were established to maintain standardized and replicable protocols regarding every step of sample handling. Molecular Medicine Ireland has published “Guidelines for Standardized Biobanking” [26]. The document outlines SOPs in detail. The authors divided SOPs into 3 sections:

- pre-clinical SOPs: Assessment of the Research Participant, Safety Guidelines, Specimen Identification and Labeling,
- clinical SOPs: Blood Collection – Venipuncture, Saliva Collection, Urine Collection, Faeces Collection, Buccal Collection, Bronchoalveolar Lavage Collection using Bronchoscopy,
- laboratory SOPs: Personal Protective Equipment, Specimen Receipt, Preparation of Serum and Plasma from Blood, DNA Extraction from Blood, RNA Extraction from Blood, Protein Extraction from Blood, Peripheral Blood Mononuclear Cell isolation from Blood, Processing of Urine, Processing of Buccal Swabs for DNA Extraction, Processing of Faeces for DNA extraction, Processing of Tissue Samples, Processing of Cultured Cells, Processing of Bronchoalveolar Lavage [26].

**Table 1:** Overview of tissue preservation and storage methods for various scientific purpose

Tissue	Target	Preservation methods (e.g. medium/buffer/vial type/kit)	Short term storage/transport conditions	Long term storage conditions	Special notes	Literature
Blood	DNA	EDTA, whole blood or serum	4 °C or on wet ice, within 24 h	−80 °C for years	–	[26–28]
	RNA	Paxgene®/Tempus™ tubes	4 °C or on wet ice, within 24 h	−80 °C for years	Blood in Tempus™ tubes may be stored up to 6 years in −80 °C.	[26, 29]
	Proteomics	Plasma separating tube with heparin and serum separation tube with heparin and Plain tube	4 °C or on wet ice, within 24 h	Plasma and RBCs in −80 °C	Storage of RBCs is recommended at −80 °C for cell membrane proteomics research.	[26–28, 30, 31]
	Biochemistry	Plasma separating tube with heparin and serum separation tub and Plain tube with heparin	4 °C or on wet ice, within 24 h	Plasma, immediate analysis or − 80 °C for years	–	[26]
	PBMC (Peripheral Blood Mononuclear Cells)	acid citrate dextrose, BD CPT™, LeukoSep™	RT, within 24 h	−80 °C or preferred LN_2_ with a cryopreservative	–	[31, 32]
	Circulating Tumor Cells	Cell-Free DNA™ BCT® tube	for at least 4 days at RT	−80 °C or preferred LN_2_ with a cryopreservative	–	[33, 34]
	Cell-free DNA	Streck® Cell-Free DNA™ Blood Collection Tubes	up to 7 days at ambient temperature, avoid shipping/storage in 4 °C	immediate extraction or − 80 °C for years	Plasma is preferable to serum, cell lysis should be avoided to prevent increase of unspecific cfDNA level. Spike-in should be considered.	[35–39]
	Circulating non-coding RNA	Plasma or plasma separating tube	4 °C or on wet ice, within 24 h	Plasma samples should be frozen immediately at −80 °C (stability) up to 1 year	Archival samples may be used	[40–42]
	Exosomes	Plasma or plasma separating tube	4 °C or on wet ice, within 24 h	−80 °C for years	Special equipment needed for exosomes isolation: ultracentrifuge/chromatographs/filters.	[43, 44]
	Platelets (e.g. tumor-educated platelets)	EDTA	4 °C or on wet ice, within 48 h	Obtain the platelet-rich plasma with series of centrifugation, process within 48 h	The platelet pellet collected on RNAlater® and frozen at −80 °C; plasma stored directly at −80 °C.	[45]
	Metabolomics	Plasma separating tube with heparin and serum separation tube and Plain tube	4 °C or on wet ice, within 24 h	−80 °C for years	–	[26] CEN/TS 16945:2016
Tumor	DNA	Tumor parts: snap freeze in cooled isopentane	Preserve within an hour from excision/biopsy; transport in closed, sterile container on ice at 4 °C before preservation	−80 °C or liquid nitrogen	–	[26, 46]
		formalin-fixed, paraffin-embedded (FFPE)	Store at RT, embed within 72 h	RT in dry conditions for years	In case of DNA extracted from FFPE samples - special NGS library preparation needed due to degraded and shredded template).	[47, 48]
	RNA	RNAlater™ - RNA preservation	Preserve within an hour from excision/biopsy; if RNA preservative added – transport within 72 h when stored at 4 °C	Discard preservation medium and store at −80 °C	May be also retrieved from FFPE samples and analyzed with NGS.	[26, 49, 50]
	Protein	Tumor part snap freeze in cooled isopentane	Preserve within an hour from excision/biopsy; transport in closed, sterile container on ice at 4 °C before preservation	store at −80 °C	–	[26]
	Microscopic morphology	FFPE – 10% buffered formalin	Store at RT, embed within 72 h	RT in dry conditions for years	Samples preserved in RNALater may also serve for microscopic morphology analysis purposes	[26, 50]
		OCT (Optimal Cutting Temperature medium)-embedded -snap frozen	Preserve within an hour from excision/biopsy; transport in closed, sterile container on ice at 4 °C before preservation	−80 °C for years		
	Cancer cells for culture	Before cells isolation – culture medium with or without fetal bovine serum (FBS)	4 °C or on wet ice, within 24 h	LN_2_ with a cryopreservative or implant in immunodeficient mouse.	Immediate dissociation of tissue is required – enzymatic dissociation (or chemical or mechanical); Specific isolation methods for different tissue [51]. Population of cancer stem cells may be separated. Different culture media additives are required for proper culturing (Table 4 in [52]).	[51–53]
Cervical cytology	DNA, RNA, protein, cells	Liquid biopsies in Thinprep (TP) containing 20 ml PreservCyt	1–4 weeks RT	−25 °C	–	[54]
Urine	Metabolic products, DNA, RNA, protein	e.g. 9 ml in the vacutainer system	4 °C or on wet ice, within 24 h	Direct storage in −80 °C or immediate analysis	–	[55, 56] CEN/TS 16945:2016
Semen	Semen analysis, DNA, RNA, protein	Sterile container	Immediate analysis: heating chamber 37 °C; for storage - 4 °C or on wet ice, within 24 h	−80 °C or preferred LN_2_ with a cryopreservative	Semen analysis - within few hours after acquisition	[57, 58]
Stool	DNA, RNA, Proteome, Microbiome	Sterile container or Genotek tubes for DNA analyses	RT, within 24 h	Direct storage in −80 °C or immediate extraction	–	[26, 59]
Saliva	DNA, biomarkers	Sterile container or collection kit	RT, within 24 h	−80 °C for years	Exceptionally increased value of specimen if provided full record of oral diseases.	[26, 60]
Breast milk	Trace of organic pollutants (POP), biochemical composition analysis	Sterile, clean bottles, e.g. with Teflon coating	4 °C, for no longer than 72 h	at −20 °C, for longer periods. Freezing expressed human milk is safe for at least 3 months.	If the sample cannot be refrigerated, you can add a small tablet of potassium dichromate (POP analysis, document available at: http://www.ccbasilea-crestocolmo.org.uy/wp-content/uploads/2010/11/ing1.final_.SOP-POP-Regional-Sampling-Breast-Milk-1.pdf date of access: 27.07.2017).	[61, 62]
Nail and hair	Metal trace, DNA, brominated and organophosphate flame retardants exposure, effects of cosmetic products	Nails should be clipped after a few weeks after recent clipping Clean, labeled envelope	room temperature in the driest condition possible	Like short term, may be stored frozen at −20 °C for long term	Limited usefulness, questionable value, noninvasive collection, status of medium/long term exposure.	[55, 63, 64]

RT – room temperature; LN_2_ – liguid nitrogen;

The wide variety of collected human tissue samples requires adequate preparation and preservation procedures for the least quality loss during transportation and storage (see Tab. 1). Even temporary storage in 0 or 4 °C exposes samples to evaporation [65]. Another often neglected issue is storage container selection, when choosing the wrong polypropylene tube type may decrease the yield of protein extraction [66]. The other essential step of pre-analytical samples’ management is to implement a replicable, precise and errorless aliquoting workflow. Except manual techniques combined with trained staff and SOPs, there are novel automated robots e.g. The Sample Array Tube Handler (Thermo Scientific, San José, CA, USA) [67]. Such solution decreases the amount of sample needed and its loss, limits human-dependent pre-analytical errors and simplifies identification and storage system. The other component of a proper identification system is labeling. There are numerous solutions like 1D, 2D barcodes, tubes with laser-etched barcodes or radio-frequency identification based labels (RFID) [68]. Regardless of labeling system type – it must be unique for each sample and its aliquot. It also should be compatible with IT system o f a Biobank to ensure fast access to the sample (decreasing thawing of other samples while searching). Unifying labeling with SPREC results in a straightforward and sharable database of a Biobank collection [69].

### IT Systems

An efficient bioinformatics in biobanks is a crucial matter. IT systems have to serve databases in a real-time, easy access and user-friendly manner. Additionally, privacy protection and anonymization components cannot be neglected (see 3.5. Ethical and legal issues subchapter). The enormous number of digital, sensitive data requires optimized both hardware and software. There has been developed international standards for data obtaining and processing to enable compatible data sharing. International organizations such as NCI, ISBER, caHUB, OECD, EC-JRC are providers of recommendation useful in harmonizing every branch of BBs’ functionality [70]. But for the neatness, a consistent database should meet the standards for the international compatibility. The issue is essential in any case of cooperation, which increases the extent and significance of a project and brings profits for the shareholders. Therefore, implementing common standards and participation in international BBs’ networks is a necessity.

Standards implementation regards every step of any information processing either biological or epidemiological. For maintaining a predictable and restorable sample collection Standard Preanalytical Coding for Biospecimens (SPREC) is recommended. This coding system enables biorepositories to describe procedures and status of just obtained sample. This simple coding has an international clear interpretation and its widespread encourages biobankers to utilize the system [71].

Compilation of SPREC, a list of data to be collected (such as BRISQ - Biospecimen Reporting for Improved Study Quality [72], NCRI recommendations) and SOPs (https://www.ctrnet.ca/operating-procedures, date of access: 27.07.2017) creates a complement and worldwide compatible system. Moreover, diligent planning and constant evolution of IT systems ensure the right maintenance of both data and samples collection along with keeping wide international collaborative capabilities (Tables 1 and 2).

**Table 2:** List of crucial guidelines concerning biobanking issues

Institution	Document	Content	Year	Source (date of access: 27.07.2017)
National Cancer Institute	NCI Best Practices for Biospecimen Resources	A. Scope, applicability, and implementation B. Technical and operational best practices C. Ethical, legal, and policy best practices	2016	https://biospecimens.cancer.gov/bestpractices/2016-NCIBestPractices.pdf
International Society for Biological and Environmental Repositories	Best Practices for Repositories: Collection, Storage, Retrieval and Distribution of Biological Materials for Research	Repository planning considerations, facilities, storage equipment and environments, quality management, safety, training, records management, cost management, biological material tracking, packaging and shipping, specimen collection, processing and retrieval, legal and ethical issues for biospecimens, specimen access, utilization and destruction	2012	http://c.ymcdn.com/sites/www.isber.org/resource/resmgr/Files/ISBER_Best_Practices_3rd_Edi.pdf
Molecular Medicine Ireland (MMI)	MMI Guidelines for Standardised Biobanking	Part I: Pre-clinical standard operating procedures Part II: Clinical standard operating procedures Part III: Laboratory standard operating procedures	2010	http://www.molecularmedicineireland.ie/wp-content/uploads/2015/09/MMIGuidelinesforStandardisedBiobanking_FINAL160610.pdf
Organization for Economic Cooperation & Development	OECD Guidelines on Human Biobanks and Genetic Research Databases	Part I. Guidelines on Human Biobanks and Genetic Research Databases Part II. Annotations	2009	https://www.oecd.org/sti/biotech/44054609.pdf

#### Record Constitution

Numerous partners provide ready-to-implement sets of data, distinguishing every piece of information is helpful for further research. NCRI has published such document, the sets of tables comprise the list, which divides information about the patient and collected samples. Each table is extended with a list of particular records. Document available at: (http://ccb.ncri.org.uk/wp-content/uploads/2014/03/CCB-Data-Standard-v1.pdf; date of access: 27.07.2017). Also, BRISQ has a resourceful and worldwide compatible protocol for preparation a novel and up-to-date database in biobank [72].

BBMRI (Biobanking and Biomolecular Resources Research Infrastructure) created in 2016 MIABIS 2.0 (Minimum Information About BIobank data Sharing) – comprehensive and detailed document describing guidelines for sharing data between biobanks within networks. It standardizes minimum information required to initiate collaboration which includes: information on biobank, sample and data collection details, study description [73]. For other relevant guidelines, see Table 2.

Biobanks may also undergo certification of their activity. NF S96–900 is a French norm legislating the requirements for the management system of a Biological Resource Centre (BRC) and the quality of biological resources. The certificate is based on OECD Guidelines and is compatible with the international ISO 9001. NF S96–900 document is available at: (http://www.p3gobservatory.org/download/NFS96-900F.pdf; date of access: 27.07.2017). The other laboratory accreditation procedure (ISO 15189) was implemented by the Swedish Cervical Cytology Biobank (SCCB) as an extension of liquid based cytology procedures at clinical laboratories. It ensures a quality management system (QMS) including quality assurance (QA) and quality control (QC) programs, covering the full spectrum of biobanking operations [54].

#### Workflow and Online Tools for Biobanking

The wide range of data, such as: demographic, ethnical, medical, environmental, genetic and other [74] collected from the patient trigger a need for the development of complex IT systems. But for the clinical data, there are sets of biological information, which also must be input to the IT system, stored and processed - thus appeared complicated tasks. Holistic IT solutions, compatible with hospital and pathology units are the optimal solution. Many researchers and companies took on the evolution of bioinformatics applications such as MIBBI, MIAME, SysMOSEEK, openBIS, Gaggle-BRM, MIMAS, XperimentR, ISA tools, BASE, LabKey [75]. One of the most interesting approaches was implemented in XTENS, which consists of a web portal, an internal database, and a data grid storage element [75]. There are a few available online tools for researchers seeking advice on legal regulations of data sharing among European countries:

- BioMedBridges Legal Assessment Tool (LAT)
- Biobanking and Biomolecular Resources Research Infrastructure (BBMRI) legal WIKI
- Human Sample Exchange Regulation Navigator (hSERN)
- International Policy interoperability and data Access Clearinghouse’ (IPAC) provided by the Public Population Project in Genomics and Society (P3G) [76].

Biobanks IT systems face further described ethical issues by administration of novel solution like short message communication with donors in case of need [77].

### Ethical and Legal Issues

Biobanks are unique units placed between a research and clinical unit. Therefore, many legal and ethical issues arose during the evolution of these novel initiatives. Despite a strong resemblance to hospital pathology units, which also store patients’ specimens, BBs have to overcome more sophisticated obstacles. These difficulties divide into groups [1]:

- Ownership

Ever since human tissue samples have been used for the R&D (Research and development) purpose, an issue of ownership claims of donors arose. Specimens may be used not only for strictly research goals (biomarkers determination, cells biology analysis and the like) but also for the discovery of new drug targets or a novel treatment. In this case, patients may become eager to participate in the results and benefits of commercialization. Until now contemporary verdicts are in favor of scientist [1]. Nevertheless, exact ownership rights or their deprivation should be established just from the startup of a biobank. Moreover, matters of ownership play a great role in transferring samples between foreign researchers (is a Material Transfer Agreement (MTA) sufficient?) along with specimen utilization or procedures after donor’s death [78].

b. Consent limitation issues

Optimization of consent content is crucial in BBs’ functioning. Appropriate consent must combine both affairs of scientist and patient. Classical informed consent turns out to be insufficient in biobanking due to the limitation of sample use for one, specific project [1, 7, 79, 80]. Additionally, there is no international consensus on the consent issue along with the differences between each legal system of each country. It hinders international sharing of samples. A novel form of consent was propagated - general/broad consent [1, 79, 80]. It comprises a patient’s agreement for the utilization of his sample for current studies and a future one (within a specified framework), without the need for the contact with the patient. But if the framework changes all the consents should be re-applied [79]. Thus, it results in wide-range, general and unspecified consents.

Along with the development of IT tools – a novel solution has been achieved, namely a dynamic consent. This type of consent requires tools for an easy accessible constant contact with the patient in order to manage re-consent for each new research [79]. But for the firm and conscious agreement, it is possible to inform patients about important finding - IF (incidental findings) and IRR (individual research results). IF is defined as “a finding concerning an individual research participant [or here, an individual contributor] that has potential health or reproductive importance and is discovered during conducting research, but is beyond the aims of the study.” [81]. Whilst, an IRR “is a finding concerning an individual contributor that has potential health or reproductive importance and is discovered in the course of research, when the finding is on the focal variables under study in meeting the stated aims of the research project” [82]. A recent study on the Australian population showed that the majority (94,4%) of queried people responded that they would like to receive “specific information obtained from your sample that may be important to your health or treatment” [83].

Biobanks storing materials obtained from children usually require parents’ (or legal guardians) consent. Ethical and legal guidelines indicate that children should be involved in the consent procedure as well. Also, child’s will: assent and dissent must be respected [84]. Another issue is whether the re-consent of pediatric patients, whose tissues in childhood were obtained and granted by parental consent, at the age of majority is needed [85]. The discussion is still ongoing [80].

c. Storage and protection of privacy-anonymization

Long-term storage of biological samples requires adequate anonymization as well as identification procedures. A vast amount of data about the specific sample and their donor needs to be protected according to best-known standards. EU Data Protection Directive obliges investigator and administrators to provide professional and safe data management with full respect to the donor sake (document available at: http://ec.europa.eu/justice/data-protection/; date of access: 27.07.2017). Data anonymization done by simply deleting an identification information is insufficient in the context of their security. Often pseudonymization is implemented (data are secured by assigning a key or cipher instead of personal data) [76]. Therefore, sample coding is done in an ordered manner, universal for all samples. Nonetheless, identification data on donor combined with a coding symbol (number/barcode) or database, enabling decoding should be managed with the highest standards. It enables researchers to contact with the donor in case of obtaining accidental and significant findings for the donor’s health [86]. Another issue, often neglected, is a case of closure of a biobank or necessity to eliminate stored samples and data. The BBs’ founders are rarely prepared for such case [87].

d. Whole genome protection and accessibility

Application of wide and accurate genomic sequencing leads to some new issues concerning security of obtained data. Storage of ones’ whole genome sequence poses a temptation for some third-parties to seize the data for their purposes. Comprehensive pieces of information encapsulated in genome sequence are utilized not only as an identification tool but also as a source of health status and burdens of the patient. Thus, thorough security procedures are implemented in every step of sample processing or analysis. Some donors are concerned about allowing BBs to process their genome information what decreases the number of volunteers [88]. However, researcher’s community opts for increasing the utility of the data by open access to genomic data along with respect to autonomy and anonymization of data. New legislation solutions, like GINA (Genetic Information Nondiscrimination Act), are needed to protect such data from misuse whilst open access [89].

Generally, any ambiguity of laws is discussed and solved by the ethics committees, which are obliged to give an opinion and requirements for a project. Currently, many countries are developing their own legal solutions according to BBMRI and/or OECD recommendations for biobank establishment.

## The Role of Biobanks in Personalized Medicine

Personalized medicine (P4) defines a new approach to a patient and the disease. The concept of this personalization comprises 4 features [25, 90]:

1. Predictive - ability to conduct fast, precise and wide analysis of risk for particular diseases requiring easy access and affordable methods. Recent intensive progression in the field brings us closer to this solution [91]. However, biobanks play a crucial role in discovering new predictive factors like genetic aberrations [14, 92, 93]. In turn, correlating the discoveries with clinical data may facilitate predicting and support next step of P4 - prevention.
2. Preventive - comprises the idea of avoiding disease progression by an early application of accurate and personalized treatment. It may not seem to be a novel concept because there are yet implemented effective preventive solutions like vaccination, but unlike vaccines, which are recommended for the majority of the population, personalized medicine focuses on individuals. Biobanks-aided advancement can bring us to the higher level. In future, it will be possible to elevate the prognostic value of early symptoms and combine them with genome data what finally will lead physicians to quick and accurate diagnosis and enable to administer the right treatment on time. Yet conducted experiments confirmed the unique role of biobanks - numerous studies presented a new risk for diseases, on the basis of data stored there [13, 94–98].
3. Personalized - genotypic and phenotypic differences in human population have a significant influence on treatment efficacy. The more individualized it is the more efficient results are obtained. Recently whole genome and whole exome sequencing are widely available and more affordable. Deep knowledge about genetic and environmental circumstances of the patient increases the accuracy of diagnosis and treatment. Biobanks are centers of both types of data [99–101].
4. Participatory - increasing awareness of both patients and medical professionals and their mutual communication are the basis of P4 medicine. Conversely, in this point importance of IT companies increases, since they mediate the patient-doctor relationship by the development of intuitive, accessible and privacy-safe-oriented systems. Moreover, bioinformatics and new IT solutions are crucial for processing and organizing huge amounts of data collected from a patient.

Each component creates a possibility for more efficient and suitable treatment choice. These units pose a chance to create a core of each part of a personalized medicine approach consistent with evidence-based medicine (EBM).

Oncologic diseases are an especial benefiter of personalized medicine solutions. In the context of P4 medicine, biobanks may significantly develop the process of prevention, diagnosis and finally the treatment dedicated to the individuals. Numerous studies prove the significant role of biobanks in mentioned steps [102–104]:

- Screening and prevention – role of PSA level in prostate cancer, circulating miR-196a and miR-196b in oral cancer [105] or chromogranin A in different malignancies e.g. ovarian cancer [106]
- Diagnosis – searching for biomarkers in pancreatic cancer [94], thyroid neoplasm [107] or in colorectal cancer [108]
- Prediction - patient response to the treatment on the basis of the genetic profile – KIT mutation (N505I) and sensitivity to imatinib [100]
- Pharmacological – patients’ reaction to the drug and proper dosage: [109–111].

## Biobanks Review among Europe

**Fig. 3:**
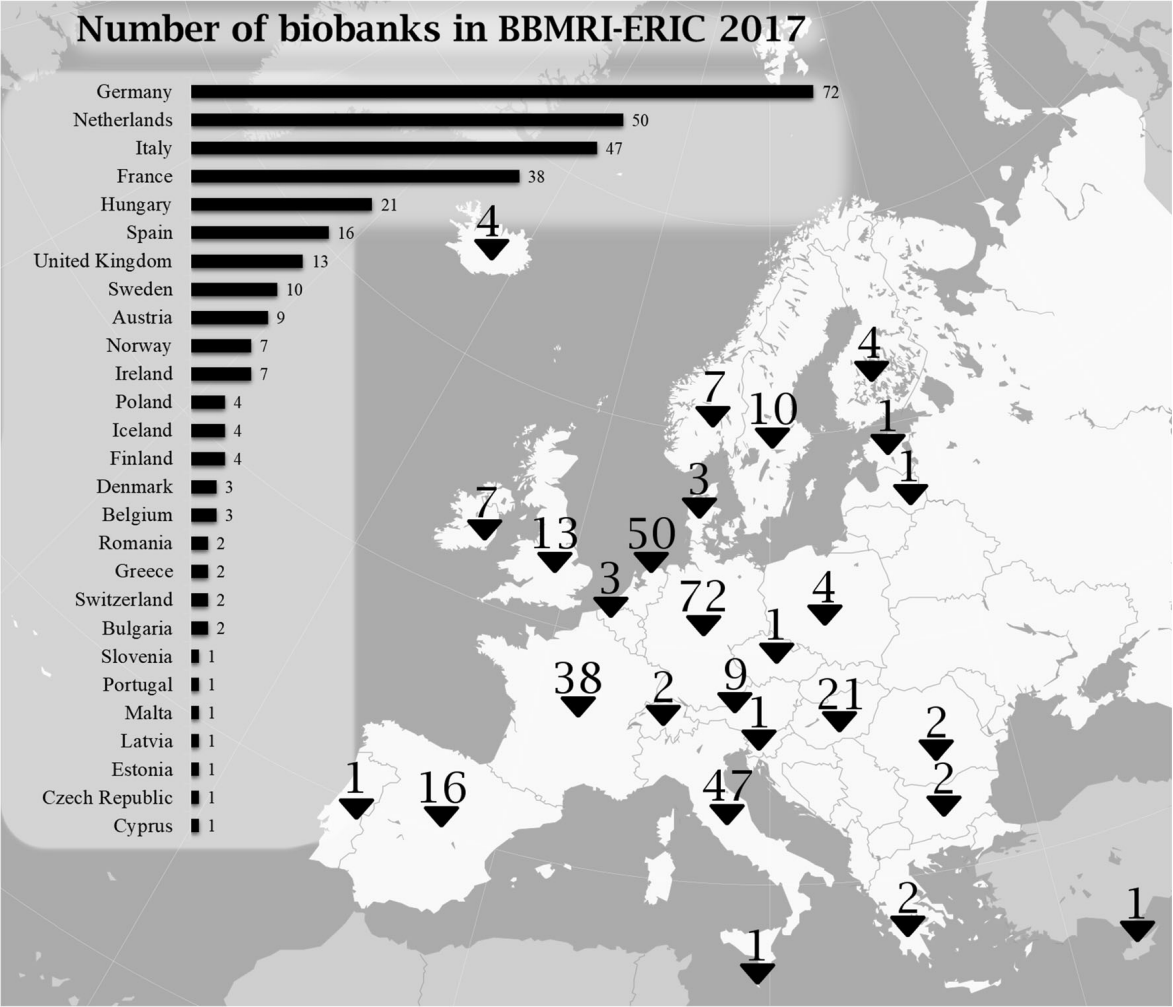
Distribution and localization of BBMRI members (July 2017, data from https://www.bbmriportal.eu; map source – Creative Commons)

Contemporarily biobanks organize themselves into international networks. Such cooperation increases the impact of research findings. BBMRI (Biobanking and Biomolecular Resources Research Infrastructure) was one of the first European Research Infrastructure projects funded by the European Commission (EC) in January 2011. For the moment, it has 325 members (Biobanks), placed in Europe (https://www.bbmriportal.eu date of access: 27.07.2017). Distribution of BBMRI members is depicted in Fig. 3 (another available https://web.bbmri-eric.eu/Directory-files/directory-map-3-1-big-labels.png, date of access 27.07.2017). In comparison, there were 636 biobanks listed in the United States of America in 2013 [112].

Another significant organization is EuroBioBank (http://www.eurobiobank.org date of access: 27.07.2017), it has 26 members: 22 biobanks (20 from Europe countries: France, Germany, Hungary, Italy, Malta, Slovenia, Spain, United-Kingdom, Turkey and 2 from Israel and Canada), EuroBioBank Coordinating Team, EURORDIS (European Organization for Rare Diseases), 3C-R: Expertise and consulting organization for biobanks, Telethon organization for genetic diseases research. EuroBioBank’s mission is to gather biological material from the patients affected by rare diseases and provide it to researchers who often have problems with obtaining enough samples. There are 130,000 samples combined with data available in the online catalogue.

## Conclusions

The role of biobanks in the constantly developing world keeps increasing over time. Even more research projects incorporate biobank establishment or utilization of samples from the BBs. Possibilities brought by these institutions enable scientists to conduct wide-scale analyses with the unparalleled extent of thoroughness and significance. However, they do not only give benefits to the scientific community, but also for a patients’ health. Lower cost of mass processing and high operation standards implementations produce benefits for the donors in the form of information about the genome, prediction and prevention advice. Constant development is supported by international networks and organizations, which share their longtime experiences and act as a guide during new biobank creation. Thus, international collaboration and support are the keys to future biobanking development. In terms of mentioned tendency, local government and legal system ought to be prepared for the forthcoming evolution of biorepositories. The interdisciplinary character of biobanks opens new pathways (and evolve the old one) for biomedical researchers, clinicians and industrial partners, which cannot be neglected.

## Abbreviations

BBs: biobanks
SOPs: Standard operating procedures
